# Early traditional Chinese medicine bundle therapy for the prevention of sepsis acute gastrointestinal injury in elderly patients with severe sepsis

**DOI:** 10.1038/srep46015

**Published:** 2017-04-06

**Authors:** Yifei Wang, Yunhua Zhang, Ronglin Jiang

**Affiliations:** 1 ICU, Zhuji Hospital of Traditional Chinese Medical Hospital, Zhuji 311800, Zhejiang Province, China; 2 ICU, Zhejiang Provincial Hospital of Traditional Chinese Medicine, Hangzhou 310000, Zhejiang Province, China

## Abstract

This study aimed to study the effect of early traditional Chinese medicine bundle therapy on the prevention of sepsis-associated acute gastrointestinal injury (AGI). This was a multicenter, prospective, observational, non-randomized cohort study of 296 consecutive patients with severe sepsis during 2013/3 and 2014/11; 150 patients received standard treatments (controls) and 146 received traditional Chinese medicine bundle therapy (intervention group) (herbal decoction gavage based on syndrome differentiation, Chinese acupuncture, application of mirabilite, and defecation mixture). D-lactic acid, diamine oxidase, endotoxin, gastrin, motilin, and intra-abdominal pressure were measured. AGI was categorized into four levels. Compared with controls, D-lactic acid, diamine oxidase, endotoxin, gastrin, and intra-abdominal pressure in the intervention group were decreased, and motilin was increased on day 7. AGI incidence in the intervention group was lower than in controls. GIF scores of the intervention AGI II and III groups were lower than in controls. The APACHE II scores of the intervention AGI II, III, and IV groups were lower than in controls. Compared with controls, mechanical ventilation time and ICU stay in the intervention group were shorter, and 28-day overall and AGI-attributed mortality were lower. For elderly patients with severe sepsis, early traditional Chinese medicine bundle therapy could decrease AGI incidence and improve prognosis.

Sepsis is a crucial clinical issue in critical care medicine. Indeed, there are approximately 750,000 cases of severe sepsis each year in the United States alone, resulting in over 200,000 deaths^1^. The immune function of patients with sepsis is depressed, potentially leading to multiple organ dysfunction syndrome (MODS)^2^.

The main manifestations of sepsis include gastrointestinal dysfunction or failure, and gastrointestinal mucosa injury, leading to the translocation of gastrointestinal bacteria and toxin and to septic shock^3^,^4^. Furthermore, aggravation of the septic shock condition will lead to the further impairment of the function of the gastrointestinal mucosa, leading to a vicious circle^3^.

Studies have shown that early enteral nutrition (EN) support may improve the nutritional status and prognosis of patients with sepsis^5^,^6^. In 2014, sepsis treatment guidelines suggested that patients with stable hemodynamics should receive EN support as soon as possible (within 48 h). This early EN support could help to maintain the integrity of the intestinal mucosa, prevent bacterial translocation and organ dysfunction, and decrease the infection rate^7^. However, in stressful situation, the function of the gastrointestinal tract is significantly weakened, resulting in difficult EN support^7^,^8^,^9^. Impaired gastrointestinal motility may cause reflux and aspiration, increasing the risk of ventilator-associated pneumonia (VAP) and leading to longer mechanical ventilation time, ICU stay, and total hospitalization. Some randomized controlled trials (RCT) found that 24–48 h of EN treatment for ICU patients with trauma could significantly decrease mortality and hospitalization costs^6^,^9^.

Treatments for sepsis include early resuscitation, anti-infection therapy, mechanical ventilation, nutritional support, immunoregulation, and symptomatic support^6^. Preventive measures for gastrointestinal dysfunction include the use of anti-acids to protect the gastrointestinal mucosal barrier, glutamine supplementation, and agents improving the microbial ecology. Based on the early goal-directed therapy (EGDT)^10^ and bundle therapy theories proposed in recent years (herbal decoction gavage based on syndrome differentiation, acupuncture, application of mirabilite, and defecation mixture)^11^,^12^,^13^, the modern Chinese medicine differentiates syndrome, pays equal attention to strengthening body resistance and eliminating pathogenic factors, and proposes the concept of traditional Chinese medicine (TCM) bundle therapy. According to the TCM theory, the six fu-organs function well when the gastrointestinal tract is unobstructed. For patients with sepsis, TCM aims to maintain unobstructed daily stool elimination, good tolerance of EN, normal borborygmus, and normal intra-abdominal pressure. Nevertheless, clinical data are still lacking about the efficiency of this approach in patients with sepsis.

Therefore, this prospective, observational, non-randomized study aimed to study the role of early traditional Chinese medicine bundle therapy on the prevention and treatment of sepsis after acute gastrointestinal injury (AGI). The patients received bundle intervention that included herbal decoction gavage according to syndrome differentiation, Chinese acupuncture at the acupoints of Stomach Meridian of Foot-Yangming, application of mirabilite on the umbilicus, and enema with defecation mixture.

## Results

### Characteristics of the patients

**Figure 1:**
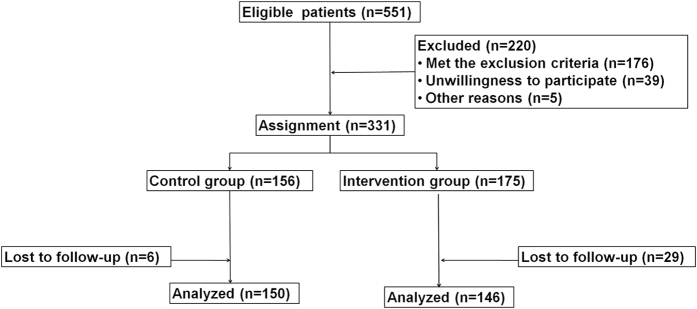
Patient flowchart.

From the 551 eligible patients, 296 patients were enrolled and included in the analysis (Fig. 1). Table 1 presents the characteristics of these patients. There were no significant differences between the two groups for age, gender, APACHE II score, SOFA score, MODS score, source of infection, and reason of ICU admission (all P > 0.05).

### Index of gastrointestinal function and intra-abdominal pressure

Table 2 presents the laboratory indexes of gastrointestinal function of the patients. On day 1, there were no significant differences between the two groups for D-lactic acid (D-lac), diamine oxidase (DAO), endotoxins, motilin (MTL), gastrin (GAS), and intra-abdominal pressure (all P > 0.05). On day 3, compared with controls, the intra-abdominal pressure was lower in the intervention group (P < 0.01) and MTL levels were higher (P < 0.05), but there were no differences for D-lac, DAO, endotoxin, and GAS (all P > 0.05). On day 7, D-lac, DAO, endotoxin, GAS, and intra-abdominal pressure were all lower in the intervention group than in controls (all P < 0.05), and MTL levels were higher (P < 0.05).

### AGI incidence

Within 7 days after admission, there were 88 patients with AGI in the control group and 69 patients in the intervention group. The incidence of AGI in the intervention group was lower than in the control group (P < 0.05) (Table 3).

### General condition of the patients

There were no differences in the distribution of patients with AGI II, III, and IV between the intervention and control groups (P > 0.05). The GIF scores of the AGI II and III subgroups of the intervention group were significantly lower than that of the AGI II and III subgroups of the control group (P < 0.05). APACHE II scores of the AGI II and III subgroups of the intervention group were lower than that of the AGI II and III subgroups of the control group (P < 0.05) (Table 4).

The patients were divided into three groups according to their APACHE II score at admission: <10, 10–20, or >20. When comparing the AGI incidence, the AGI incidence of the <10 and 10–20 subgroups in the intervention group were lower than in the <10 and 10–20 subgroups of the control group (P < 0.05) (Table 5).

**Figure 2:**
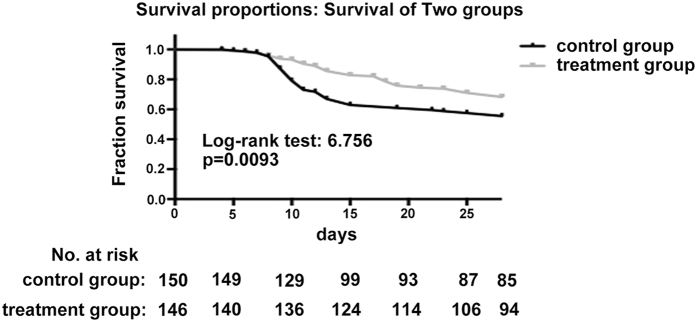
28-day survival curve between the control and intervention groups.

The mechanical ventilation time and ICU stay in the intervention group were significantly shorter than in the control group (both P < 0.05). The 28-day and AGI-related mortality in the intervention group were lower than in the control group (P < 0.05) (Table 6 and Fig. 2).

## Discussion

The aim of the present study was to study the role of early traditional Chinese medicine bundle therapy on the prevention and treatment of sepsis-associated AGI. Results showed that compared with controls, D-lac, DAO, endotoxin, gastrin, and intra-abdominal pressure in the intervention group were decreased on day 7. MTL was increased on day 7. AGI incidence in the intervention group was lower than in controls. GIF scores of the intervention AGI II and III groups were lower than in controls. The APACHE II scores of the intervention AGI II, III, and IV groups were lower than in controls. Compared with controls, mechanical ventilation time in the intervention group was shorter, the ICU stay was shorter, and the 28-day overall and AGI-attributed mortality were lower.

Severe is chemia and anoxia observed in patients with sepsis often lead to gastrointestinal mucosal barrier dysfunction, allowing intestinal bacteria and endotoxin translocation into the systemic circulation. In the last decade, critical disease monitoring and organ function support technologies improved greatly, but mortality from sepsis is still as high as 20–50%^1^. The therapeutic approach of western medicine on gastrointestinal dysfunction induced by sepsis is still limited to symptomatic treatment. Some randomized clinical trials showed that in the first 24–48 h, EN treatment for patients with critical illness could significantly decrease mortality and hospitalization costs of patients in the ICU^8^,^14^. Reintam *et al*.^15^ suggested that because of the complex and various functions of the gastrointestinal tract, a single treatment would not improve the gastrointestinal tract in cases of severe illness.

On the other hand, the spleen-stomach theory of TCM pays attention to the spleen-stomach function, which is a crucial principle of syndrome differentiation. In the Treatise on the Spleen and Stomach, Li^16^ indicated that internal impairment of the spleen and stomach will cause various diseases. TCM believes that there cannot be any survival without stomach Qi.

TCM bundle therapy includes nasal feeding, tongbian mixture enema, umbilicus compress of mirabilite, Chinese acupuncture, and moxibustion. According to the TCM theory, gastrointestinal dysfunction induced by sepsis can be divided into four types: Qi activity stagnation (gastrointestinal mucosal disorders with weakened gastrointestinal motility and impaired blood circulation in abdominal organs), Qi stagnation and blood stasis (increased capillary permeability, platelet aggregation and adhesion, and blood viscosity, leading to decreased blood flow and impaired local blood circulation), asthenia of the spleen and stomach (inhibition of the hypothalamic pituitary adrenal axis leading to excessive tumor necrosis factor synthesis and release, resulting in energy metabolism dysfunction, reduced immune function, and infection spread, therefore accelerating the development of MODS), and dampness and heat from the interior (increased endotoxin release and reduced body’s ability to scavenge free radicals, leading to excessive inflammatory reaction and organ injury)^17^,^18^. These syndromes are treated with Simo Yinzi, taohe chengqitang, six gentlemen decoction, and peptic powder, respectively.

The main component of tongbian mixture enema is *Rheum officinale*, which have various pharmacological actions and can protect the gastrointestinal mucosal barrier, promote gastrointestinal motility^19^, alleviate inflammatory reaction, decrease intestinal permeability, inhibit the absorption of endotoxin and growth of pathogenic bacterium, and improve cellular immunity and proliferation of lymphocyte to prevent GIF^20^. The main components of mirabilite are sodium sulfate, calcium sulfate, and magnesium sulfate. Mirabilite has strong water absorption power, some effects against diarrhea and detumescence, and diuretic effects. External application of mirabilite on the abdomen can withdraw the ascites, ease the burden of abdominal distension and intestinal tract, promote the detumescence of enterocoelic swollen organs, and is beneficial to the control of inflammation and recovery of intestinal functions^21^.

Acupuncture at the acupoints of Stomach Meridian of Foot-Yangming has some effects including regulating the spleen and stomach, invigorating spleen-reinforcing Qi, clearing and activating the channels and collaterals, strengthening body resistance and eliminating evil, dual-direction regulation of nerve-internal secretion-immune systems, inhibition of systemic inflammatory response, and few adverse reactions^22^. A previous study showed that acupuncture at ST36 and EA could improve the intestinal mucosal barrier in sepsis^13^.

DAO is an enzyme with high activity that exists in the intestinal mucosal epithelium. Under pathological states, intestinal mucosal cells suffer from an ischemic injury, and DAO is released into circulation; it is therefore a marker of intestinal damage. D-lac is a metabolite of intestinal bacteria fermentation. Endotoxin is a direct marker of the presence of Gram-negative intestinal bacteria. Normally, D-lac and endotoxins cannot get through the intestinal mucosal barrier; therefore, any increase of their blood levels indicates changes of intestinal permeability or barrier function, and D-lac and DAO are early diagnosis indicators of gastrointestinal mucosal barrier function dysfunction^23^. The present study showed that on the 3^rd^ day after admission, the levels of D-lac, DAO, and endotoxin in the intervention group were lower than in the control group, but without reaching statistical significance (P > 0.05). But on the 7^th^ day, levels of D-lac, DAO, and endotoxin in the intervention group were significantly lower than in the control group, indicating that gastrointestinal mucosal barrier function dysfunction was alleviated.

MTL and GAS are key gastrointestinal hormones involved in gastrointestinal motility. MTL is an excitant gastrointestinal hormone that can regulate the cyclic activity of interdigestive myoelectric complexes, maintain normal peristalsis of the stomach and small intestine, and prevent the over-growth of gastrointestinal bacteria^24^. GAS is secreted by G-cells in the antrum and duodenum, and can promote gastric acid secretion. In patients with severe sepsis who are in a stressed state, the gastrointestinal blood supply is reduced, acid-base balance is impaired, GAS secretion increases, and stress ulcer hemorrhage is easily induced. The present study showed that on the 3^rd^ and 7^th^ days after admission, MTL levels in the intervention group were higher than in the control group, and that GAS levels in the intervention group were lower than in the control group, strongly suggesting that TCM bundle therapy could significantly improve the secretion of gastrointestinal hormones in patient with severe sepsis and should help maintaining the gastrointestinal functions.

In recent years, some studies demonstrated that acupuncture could stimulate the release of peptidergic neurotransmitters, including MTL, and activate the peptidergic neural pathway to promote the recovery of inhibited gastric myoelectric activity^25^. The present study also found that on the 3^rd^ and 7^th^ days, the intra-abdominal pressure in the intervention group was obviously lower than in the control group and that the incidence of abdominal compartment syndrome was decreased, leading to improved gastrointestinal function of patients with severe sepsis.

The present study showed that after TCM bundle intervention, the AGI incidence over 7 days was significantly decreased. GIF and APACHE II scores of patients with AGI II and III in the intervention group were lower than in the control group, but without difference in AGI IV. Therefore, TCM bundle therapy could effectively improve light and moderate gastrointestinal dysfunction. But with aggravating disease, the factors influencing gastrointestinal function are increased and probably overcome the effects of TCM. Thus, the present study indicated that there were no differences of GIF and APACHE II scores among patients with AGI IV. TCM treatments should be studied further to determine more effective approaches.

Fang *et al*.^26^ found that the APACHE II, SOFA, and GIF scores of patients with MODS receiving *Rheum officinale* were obviously lower than in the standard group, and that their prognosis was better. In the present study, TCM bundle therapy was used to prevent AGI and good effects were achieved, as reflected by the lower GIF and APACHE II scores. After analysis of patients with various APACHE II scores, the study showed that TCM was more effective for patients with low APACHE II scores at admission. Zhang *et al*.^27^ considered that AGI levels were significantly associated with 28-day mortality and APACHE II score. In the present study, compared with controls, APACHE II scores in the intervention group were lower, mechanical ventilation time and stay in ICU were shorter, and 28-day mortality and gastrointestinal-related mortality were lower. These data strongly suggest that TCM bundle intervention could reduce disease severity of patients with severe sepsis. A recent meta-analysis revealed that addition of TCM added benefits in patients with sepsis^12^.

The present study was not designed to examine the exact mechanisms of TCM bundle therapy because of the complexity of the TCM mixtures. Nevertheless, some animal studies could help shed some light on these mechanisms. Indeed, Gou *et al*.^28^ showed that TCM could decrease 5-FU-induced intestinal mucositis through reduced apoptosis and necrosis of the intestinal epithelium via the suppression of inflammatory cytokine upregulation. Kim *et al*.^20^ showed that TCM bundle therapy has beneficial effects on the GI tract through modulating pacemaker potentials via 5-HT3 and 5-HT4 receptors and Ca^2+^ flux; in addition, the MAPK pathway is involved. Combinations of TCM with acupuncture have been shown to be beneficial against chronic GI disorders, as reviewed by Lee *et al*.^29^. Finally, a study showed that electroacupuncture could protect the intestinal mucosal immune barrier in rat models of sepsis^13^. Although these results are limited, they nevertheless provide some clues about the mechanisms of TCM on sepsis AGI, but additional studies are necessary to better understand these mechanisms.

The present study is not without limitations. Indeed, the sample size was small and the patients were highly selected. Furthermore, even if a number of studies have shown the beneficial effects of TCM, the exact mechanisms of action are still mostly unknown. The treatment lasted only 7 days, but it was considered to be the acute phase of AGI. Follow-up was only of 28 days because of the acute nature of sepsis AGI. Additional studies are necessary to characterize TCM and improve its efficacy.

In conclusion, for elderly patients with severe sepsis, early TCM bundle therapy could help avoiding AGI and improving prognosis.

## Methods

### Study design

This was a prospective, observational, non-randomized, multicenter study of 551 consecutive patients with severe sepsis treated at the ICUs of the Zhejiang Provincial Hospital of Traditional Chinese Medical, Tongde Hospital of Zhejiang Province, Xinhua Hospital of Zhejiang Province, and Hangzhou Traditional Chinese Medical Hospital during March 2013 and November 2014. The study protocol was approved by the Ethics Committee of each participating hospital. Informed consent forms were signed by the legal representatives of the patients. All procedures were performed in accordance with the relevant guidelines and regulations. The study was registered with the Chinese Clinical Trial Registry (Registration number: ChiCTR-IOR-15007625; Registration date: 2015-11-18; Available online: http://www.chictr.org.cn/showproj.aspx?proj=12504).

### Patients

All patients were diagnosed with severe sepsis according to the diagnostic criteria of the International Guidelines for Management of Severe sepsis and Septic Shock 2012^1^. The patients had to meet any of the following criteria^30^ and be accompanied with organ dysfunction and/or tissue hypoperfusion caused by sepsis: 1) hypotension caused by sepsis; 2) lactic acid levels higher than the normal value; 3) although given enough fluid resuscitation, urine volume remains <0.5 ml/kg/h for at least 2 hours; 4) non-pneumonic acute lung injury and PaO2/FiO2 <200 mmHg; 5) pneumonic acute lung injury and PaO2/FiO2 <200 mmHg; 6) serum creatinine >176.8 μmol/L (2.0 mg/dl); 7) bilirubin >34.2 μmol/L (2.0 mg/dl); (8) platelets <100,000/μL; and/or 9) blood coagulation disorders (international normalized ratio (INR) >1.5).

The exclusion criteria were: 1) ICU stay <3 days; 2) ≤18 years of age; 3) pregnant or lactating women; 4) terminal malignant tumor; 5) Marshall score ≥20; 6) esophagus, stomach, or intestinal medical and surgical histories or primary injury to the gastrointestinal tract when admitted to the ICU; 7) with AGI at admission; or 8) unstable hemodynamics or significantly abnormal coagulation function after 2 days at the ICU that cannot be treated with TCM nasal feeding (APTT > 2 times the upper limit of normal).

### Treatment allocation

Patients with stable hemodynamics (mean arterial pressure (MAP) ≥65 mmHg) were immediately assigned to one of two groups, based on the willingness of the legal representative of the patients to receive TCM: 156 patients did not receive TCM (controls) and 175 received TCM (intervention group). The patients in the intervention group received TCM bundle therapy in addition to standard of care. Patients in the control group received the standard of care.

### Treatments in the control group

In the control group, according to the International Sepsis Treatment Guide 2012, all patients received early EGDT for shock patients, respiratory and circulation support, active anti-infection therapy, and maintenance of electrolyte and acid-base balance. Cases with stable hemodynamics received early EN support^31^,^32^ as soon as possible. A nasogastric tube or naso-intestinal tube was indwelled in each patient. EN was performed with continuous intravenous infusion using a nutrition pump. Parenteral nutrition (PN) support within a week was avoided as much as possible.

### Treatments in the TCM bundle therapy group

In the intervention group, the patients received TCM bundle therapy in addition to the treatments of the control group. Two physicians with the level of deputy director or director diagnosed the TCM syndrome differentiation according to the Diagnostics of Traditional Chinese Medicine^33^. The diagnostic criteria for Qi activity stagnation are: swelled and painful abdomen; nausea; vomiting; constipation; red tongue; thin white moss; string; and rapid pulse. For Qi stagnation and blood stasis, the diagnostic criteria are: dusky face; sharp lancinating pain in certain locations; dry mouth but no desire for drink; dark purple tongue with ecchymotic spots on the surface; and uneven, slow, and deep pulse, or deep and thready pulse. For asthenia of the spleen and stomach, the criteria are: patients with pale face; severe dyspnea and tachypnea; lethargy and obfuscation with no desire for eating; perfuse perspiration; cold limbs; pale tongue; white moss; and extremely weak and deep pulse. Finally, the diagnostic criteria for dampness and heat from the interior are: patients with continuous high fever; restlessness and agitation; confusion of the mind; nausea and vomiting; sticky sweat; convulsion; red tongue; yellow and greasy moss; and soft and rapid pulse.

Qi activity stagnation (n = 45) was treated with Simo Yinzi. Qi stagnation and blood stasis (n = 36) was treated with taohe chengqitang. Asthenia of spleen and stomach (n = 35) was mainly treated with the six gentlemen decoction. Dampness and heat from interior (n = 30) was mainly treated with peptic powder. All decoctions were prepared in a dedicated TCM laboratory. The patients received one dose (100 mL) each day, by nasal feeding, in two doses of 50 mL. If the syndrome type changed during treatment, the treatment was adjusted accordingly. All decoctions were prepared using an YHD20-GL medical decoction system (Beijing Dongshua Medical Equipment Co., Ltd., Beijing, China). The ingredients were weighed in a non-woven bag and soaked for 30 min. The bag was removed for 30 min and then squeezed to obtain all liquid. The decoction was filtered and the volume was adjusted to 100 ml.

The recipes of the four TCM mixtures were 1) Simo Yinzi: 6 g of *Lignum aquilariae sinensis*, 6 g of root of combined spicebush root, 6 g of south common vladimiria root, and 6 g of *Fructus aurantii*; 2) Taohe chengqitang: 12 g of *Semen persicae*, 12 g of *Rheum officinale*, 12 g of prepared licorice root, 6 g of cassia twig, and 6 g of mirabilite; 3) six gentlemen decoction: 6 g of ginseng, 12 g of bighead atractylodes rhizome, 6 g of *Poria cocos*, 4 g of liquorice, 5 g of orange peel, 6 g of roasted *Pinellia ternata*, 5 g of *Fructus amomi*, and 4 g of *Radix aucklandiae*; and 4) peptic powder: 12 g of *Rhizoma atractylodis*, 9 g of *Mangnolia officinalis*, 6 g of orange peel, and 3 g of liquorice.

The selection of the decoctions was based on the available literature. According to the Science of Chinese Materia Medica manual^34^, *Lignum aquilariae sinensis* has spasmolysis effect on the gastrointestinal smooth muscle. Root of combined spicebush root can facilitate gastrointestinal motility, relieve gastrointestinal spasm, and inhibit anabrosis. South common vladimiria root can slightly stimulate the intestinal tract and significantly improve the tonicity and rhythmicity. *Fructus aurantii* can increase the gastrointestinal motion frequency. *Semen persicae* has anticoagulation effect and can improve hemodynamics. *Rheum officinale* has some effects including diarrhea effect with dual function of activating and inhibiting the gastrointestinal tract, cholagogic effect, liver protection, promotion of pancreatic secretion, inhibition of pancreatic enzyme activity, anti-ulcer, and anti-duodenal ulcer effects. Prepared licorice root has some effects including anti-ulcer effect, inhibition of gastric acid secretion, and relief of gastrointestinal smooth muscle spasms. Cassia twig cause gastrointestinal hyperperistalsis and eliminate the corrupt gas in the intestines. Mirabilite contains Na_2_SO_4_·10 H_2_O. After administration, the sulfate ion is not easily absorbed by intestinal mucosa and can form hypertonic solution that will increase the enteral fluid retention, leading to mechanical stimulation as well as intestinal peristalsis. Ginseng can improve gastrointestinal, cardiopulmonary, and immunological functions. Bighead atractylodes rhizome can adjust the gastrointestinal function and enhance the immunologic function. Poria cocos can relieve gastrointestinal spasms, inhibit gastric acid secretion, and enhance immunological function. Orange peel can promote the secretion of salivary amylase, gastric emptying, and intestinal movement. Roasted *Pinellia ternata* has some effects including antitussive, emetic, antemetic, anticancer, and cholagogue effects that adjust the gastrointestinal function. *Fructus amomi* can promote gastric emptying and intestine transmission. *Rhizoma atractylodis* can adjust gastrointestinal motility, inhibit gastric acid secretion and enhance the protective effect of gastric mucosa. *Mangnolia officinalis* can adjust gastrointestinal motility, inhibit secretion of digestive juice, and have anti-ulcer effect. Nevertheless, it has to be noted that these formulations are used in TCM for a variety of diseases and were not designed specifically for the present study.

The patients in the intervention group were treated with Chinese acupuncture at the acupoints of Stomach Meridian of Foot-Yangming: Zusanli (ST36, bilateral), Tianshu (bilateral), Shangjuxu (bilateral), and Xiajuxu (bilateral). These acupoints were selected based on the literature^13^,^35^ and the clinical experience of the physicians. The stomach meridian acupoint of the Foot-Yang ming is closely related to the gastrointestinal functions such as regulating gastrointestinal motor function, balancing gastrointestinal hormones, and protecting the gastric mucosa^13^,^35^. The location standard of the acupoints referred to the National Standard Nomenclature and Location of Acupuncture Points 2006 (GB/T12346-2006). The needles used were 0.35 × 75-mm stainless steel filiform needles (Wujiang Jia Chen Acupuncture Instrument Co., Ltd, Jiangsu, China). The operating physician had to be certified in Chinese acupuncture and to have at least 5 years of experience. The sterilized acupuncture needles were used to perform perpendicular needling. The acupuncture methods were based on Deqi and were manual. Shallow needling was performed for patients with higher muscular tension, with the manipulation of holding, twisting, lifting, and thrusting with a small amplitude and fast frequency. The needle was retained after developing needle sensation. Normal acupuncture treatment was performed for patients with low needle sensation, with even and slow manipulation of holding, twisting, lifting, and thrusting after needling to a certain depth. The needle was retained for 30 minutes after developing needle sensation, one time a day, for 7 days. Acupuncture was not performed for patients with local skin infection and anabrosis around the acupoints. Mirabilite and laxative were applied simultaneously with acupuncture.

For external application of mirabilite to the abdomen, 500 g of mirabilite were mashed into granules and put in a sand bag (about 25 × 20 cm). The sand bag was spread to the middle and upper abdomen. The bag was replaced when it became hard or crystallized. The bag was applied three times per day, 2 hours each time.

The defecation drug mixture was made by the Zhejiang Provincial Hospital of Traditional Chinese Medical. Each liter of mixture contained 8.2 g of Chinese honey locust and 160 g of honey. The mixture (50 mL) was slowly infused in the rectum and retained for 20 min, once a day. This was not performed for patients with severe diarrhea.

### Data collection and laboratory examinations

Age, gender, source of infection, and reason of ICU admission were collected at admission. Sequential organ failure assessment (SOFA), APACHE II, and MODS occurring within 24 h of admission were recorded. Blood was sampled on the 1^st^, 3^rd^, and 7^th^ days after admission to determine the levels of D-lac, DAO, endotoxin, MTL, GAS, and intra-abdominal pressure. The 28-day survival was collected.

D-lac, DAO, endotoxin, MTL, GAS, prealbumin, and albumin levels were determined at a third level class A hospital. DAO was determined by spectrophotometry (Nanjing Jiancheng Bioengineering Institute, Nanjing, China). D-lac was determined using modified enzymatic spectrophotometry (Nanjing Jiancheng Bioengineering Institute, Nanjing, China). The quantitative determination of endotoxin was performed with a limulus kit (Sigma, St Louis, MO, USA). GAS was determined with the GAS radioimmunoassay kit (China Institute of Atomic Energy, Beijing, China). MTL was determined with the motilin radioimmunoassay kit (Shanghai Kerui Biotechnology Center, Shanghai, China). GAS and MTL were determined with ELISA.

The amount of EN amount was determined as the fed EN solution volume minus the gastric residual volume, which was determined by extracting the gastric residual volume every 6 h.

Foley catheters were connected with a urine collection bag and piezometer tube with the patients in the supine position. The bladder was emptied. The urine collection bag was closed and filled with 20 mL of sodium chloride (0.9%) through the piezometer tube. The piezometer tube and transducer were connected, the distal end was open, and after zero adjustment with the pubic symphysis taken as the zero, the intravesical pressure was determined as the reference for intra-abdominal pressure.

AGI, as proposed by the European Society Intensive Care Medicine (ESICM) in 2012^36^, indicates gastrointestinal dysfunction caused by acute illness of patients with critical illness. Depending on the severity, AGI could be divided into four levels: Level I indicates that there are risks of gastrointestinal dysfunction (GID) and temporary gastrointestinal symptoms with definite reasons; Level II indicates GID^37^ in which gastrointestinal digestion and absorption functions cannot satisfy the need for nutrient and water absorption, without the influence on the general physical condition of the patient; Level III indicates gastrointestinal failure (GIF), in which the gastrointestinal function fails to recover and the general physical condition is not improved although some intervention treatments are taken; and Level IV indicates that GIF and other organs are severely affected, with MODS and shock. Due to the reversibility and spontaneous recovery of patients with AGI I, the patients with AGI II, III, and IV were grouped as being ill. Reintam *et al*.^38^ proposed three typical gastrointestinal syndromes of GIF: feeding intolerance syndrome (FI), gastrointestinal hemorrhage (GIH), and paralytic ileus (PI).

According to the standard for GIF evaluation by Reintam *et al*.^39^ and Sun *et al*.^40^, 0 indicates that the gastrointestinal function is normal; 1 indicates that the EN amount is less than 50% of the necessary amount or the patients cannot take food 3 days after abdominal operation; 2 indicates gastrointestinal nutrition intolerance or intra-abdominal hypertension; 3 indicates gastrointestinal nutrition intolerance with intra-abdominal hypertension; and 4 indicates abdominal compartment syndrome.

### Adverse events

If an adverse event was suspected of resulting from the TCM intervention, the investigator had to fill the form for adverse events and drop the patient out of the study.

### Statistical analysis

Continuous data was presented as mean ± standard deviation and analyzed with the independent-samples t test or ANOVA with the Tukey’s post hoc test, as appropriate. Categorical data was presented as frequencies and analyzed with the Fisher exact test. Survival was analyzed according to the Kaplan-Meier method and analyzed with the Log rank test. Statistical analysis was performed with SPSS 21.0 (IBM, Armonk, NY, USA). Two-sided P-values < 0.05 were considered statistically significant.

## Additional Information

**How to cite this article:** Wang, Y. F. *et al*. Early traditional Chinese medicine bundle therapy for the prevention of sepsis acute gastrointestinal injury in elderly patients with severe sepsis. *Sci. Rep.* **7**, 46015; doi: 10.1038/srep46015 (2017).

**Publisher's note:** Springer Nature remains neutral with regard to jurisdictional claims in published maps and institutional affiliations.

## Figures and Tables

**Table 1:** Characteristics of the 296 patients with sepsis.

	Control group (n = 150)	Intervention group (n = 146)	P
Age (years)	76.4 ± 13.9	76.5 ± 14.0	0.927
Male, n (%)	109 (72.7%)	109 (74.7%)	0.792
SOFA score	8.72 ± 1.51	9.02 ± 1.40	0.081
APACHE II score	15.5 ± 2.0	15.9 ± 2.0	0.085
Original infection sites, n (%)			
Respiratory system	80 (53.3%)	76 (52.1%)	0.787
Digestive System	37 (24.7%)	29 (19.9%)	
Urinary system	7 (4.7%)	10 (6.9%)	
Hematogenous infection	9 (6.0%)	12 (8.2%)	
Intracranial infection	5 (3.3%)	4 (2.7%)	
Other sites	12 (8.0%)	15 (10.3%)	
Reason of ICU admission, n (%)			
Respiratory failure	69 (46.0%)	75 (51.4%)	0.918
Circulatory failure	20 (13.3%)	18 (12.3%)	
Nervous system (epilepsy, apoplexy)	21 (14.0%)	16 (11.0%)	
Trauma	17 (11.3%)	18 (12.3%)	
Others	3 (2.0%)	2 (1.4%)	
Postoperative patient	20 (13.3%)	17 (11.6%)	
Traditional Chinese medicine syndrome			
Qi activity stagnation	52 (34.7%)	45 (30.8%)	0.773
Qi stagnation and blood stasis	40 (26.7%)	36 (24.7%)	
Spleen-stomach weakness	30 (20.0%)	35 (24.0%)	
Internal accumulation of damp-heat	28 (18.7%)	30 (20.6%)	

**Table 2:** Laboratory indexes of gastrointestinal function.

Indexes	Time point	Control group (n_1_ = 150) (n_3_ = 150) (n_7_ = 136)	Intervention group (n_1_ = 146) (n_3_ = 146) (n_7_ = 138)	P
D lactic acid (mg/L)	Day 1	508.25 ± 285.03	506.54 ± 279.74	0.960
	Day 3	392.21 ± 186.26	373.01 ± 163.78	0.406
	Day 7	377.79 ± 204.93	321.65 ± 138.78	0.044
Diamine oxidase (U/mL)	Day 1	12.98 ± 7.31	12.97 ± 7.31	0.992
	Day 3	10.65 ± 5.32	11.11 ± 5.84	0.530
	Day 7	12.36 ± 6.43	10.09 ± 4.55	0.010
Endotoxin (eU/ml)	Day 1	0.77 ± 0.23	0.78 ± 0.21	0.630
	Day 3	0.68 ± 0.18	0.63 ± 0.19	0.097
	Day 7	0.64 ± 0.17	0.59 ± 0.17	0.035
Motilin (ng/L)	Day 1	273.05 ± 198.18	268.55 ± 198.18	0.844
	Day 3	274.08 ± 165.02	324.12 ± 201.30	0.041
	Day 7	320.54 ± 230.24	423.02 ± 318.69	0.021
Gastrin (ng/L)	Day 1	221.82 ± 177.96	216.42 ± 177.63	0.794
	Day 3	216.24 ± 120.75	183.79 ± 113.08	0.041
	Day 7	214.32 ± 121.37	176.54 ± 99.14	0.037
Intra-abdominal pressure (mmHg)	Day 1	9.77 ± 4.02	10.30 ± 2.86	0.193
	Day 3	11.23 ± 4.11	9.16 ± 4.12	< 0.001
	Day 7	11.59 ± 3.80	10.28 ± 2.61	0.012

Note: n_1_, n_3_, and n_7_ represent the number of surviving patients on the 1^st^, 3^rd^, and 7^th^ days.

**Table 3:** Comparison of AGI incidence.

Groups	Control group (n = 150)	Intervention group (n = 146)	P
AGI (%)	88 (58.67%)	69 (47.26%)	0.041
No AGI (%)	62 (41.33%)	77 (52.74%)	

**Table 4:** General condition of the patients.

	Control group (n_7_ = 88)	Intervention group (n_7_ = 69)	P
AGI II			
n	13 (14.8%)	18 (26.1%)	0.077
GIF score	2.7 ± 1.0	2.2 ± 0.9	0.006
APACHE II score	12.2 ± 2.5	11.3 ± 2.0	0.011
AGI III			
n	68 (77.3%)	48 (69.6%)	0.275
GIF score	2.9 ± 1.0	2.4 ± 1.0	0.007
APACHE II score	11.1 ± 2.0	10.2 ± 1.9	0.013
AGI IV			
n	7 (8.0%)	3 (4.4%)	0.358
GIF score	4.7 ± 0.7	4.6 ± 0.8	0.552
APACHE II score	13.7 ± 1.9	13.2 ± 1.6	0.023

**Table 5:** Comparison of the AGI incidence according to APACHE II scores.

	APACHE II < 10, n (AGI incidence)	APACHE II 10–20, n (AGI incidence)	APACHE II > 20, n (AGI incidence)
Control group (n = 150)	24 (25.0%)	70 (51.4%)	56 (82.1%)
Intervention group (n = 146)	21 (0%)	67 (31.3%)	58 (82.8%)
P	0.023	0.024	1.000

**Table 6:** Comparison of prognosis between the two groups.

Parameters	Control group	Intervention group	P
	(n = 150)	(n = 146)	
Mechanical ventilation time (days)	10.4 ± 3.7	9.3 ± 3.8	0.011
ICU stay (days)	18.5 ± 10.6	14.2 ± 7.9	0.035
28-day mortality	43.3%	31.5%	0.048
AGI-attributable mortality	8.7%	2.7%	0.043
